# When Eyes Wander Around: Mind-Wandering as Revealed by Eye Movement Analysis with Hidden Markov Models

**DOI:** 10.3390/s21227569

**Published:** 2021-11-14

**Authors:** Hsing-Hao Lee, Zih-Ling Chen, Su-Ling Yeh, Janet Huiwen Hsiao, An-Yeu (Andy) Wu

**Affiliations:** 1 Department of Psychology, College of Science, National Taiwan University, Taipei City 10617, Taiwan; hsinghaolee@nyu.edu; 2 Graduate Institute of Brain and Mind Sciences, College of Medicine, National Taiwan University, Taipei City 10051, Taiwan; r06454014@ntu.edu.tw; 3 Neurobiology and Cognitive Science Center, National Taiwan University, Taipei City 10617, Taiwan; 4 Center for Artificial Intelligence and Advanced Robotics, National Taiwan University, Taipei City 10617, Taiwan; 5 Center for Advanced Study in the Behavioral Sciences, Stanford University, Stanford, CA 94305, USA; 6 Department of Psychology, The University of Hong Kong, Pok Fu Lam, Hong Kong; jhsiao@hku.hk; 7 State Key Laboratory of Brain and Cognitive Sciences, The University of Hong Kong, Pok Fu Lam, Hong Kong; 8 Graduate Institute of Electronics Engineering, National Taiwan University, Taipei City 10617, Taiwan; andywu@ntu.edu.tw

**Keywords:** mind-wandering, sustained attention, eye movement analysis with hidden Markov models (EMHMM), fixation, learning

## Abstract

Mind-wandering has been shown to largely influence our learning efficiency, especially in the digital and distracting era nowadays. Detecting mind-wandering thus becomes imperative in educational scenarios. Here, we used a wearable eye-tracker to record eye movements during the sustained attention to response task. Eye movement analysis with hidden Markov models (EMHMM), which takes both spatial and temporal eye-movement information into account, was used to examine if participants’ eye movement patterns can differentiate between the states of focused attention and mind-wandering. Two representative eye movement patterns were discovered through clustering using EMHMM: centralized and distributed patterns. Results showed that participants with the centralized pattern had better performance on detecting targets and rated themselves as more focused than those with the distributed pattern. This study indicates that distinct eye movement patterns are associated with different attentional states (focused attention vs. mind-wandering) and demonstrates a novel approach in using EMHMM to study attention. Moreover, this study provides a potential approach to capture the mind-wandering state in the classroom without interrupting the ongoing learning behavior.

## 1. Introduction

Mind-wandering (MW), the shift of attention from the current task to task-unrelated thoughts, is a universal experience that occupies 47% of adults’ daily thinking time [1]. We live in an era full of distractions where modern technology and social media have become a pervasive part of our lives. The increase of distractions has caused people to have more difficulty in concentrating on tasks. Although MW benefits creativity, imagination, and plans for the future [2,3], it also accompanies negative emotional feelings [4]. Moreover, MW is negatively correlated with task performance. For example, Stothart et al. [5] showed that cellphone notifications disrupted task performance in an attention demanding task, even when participants did not check their phones. Other studies also showed that MW impaired the extent of text comprehension [6,7], and even jeopardized safety during driving [8]. In the educational scenario, MW can impact learning efficiency [9] and yield significant class performance cost [10]. Therefore, understanding when and what kind of people tend to mind-wander is a critical issue in modern society [11].

Detecting MW using a wearable device can help resolve this issue, and viewers’ eye movements can be used as a good index of their attentional states. Indeed, as remote classes have become more mainstream recently due to the COVID-19 pandemic, one of the biggest challenges of online learning is struggling with staying focused on a screen for long periods of time. With online learning, there is also a higher chance for students to be distracted by social media, advertisements, or other websites. An eye-tracking detection system could thus be an alternative strategy for supervision without directly interfering with classes. By applying a wearable eye-tracking system for detecting attentional states, it is possible for instructors to notice when students have lapses of attention and adjust content accordingly. Additionally, with the development of imaging processing techniques, it is possible to capture people’s eye movements using a low-cost camcorder (e.g., [12]), which makes it more plausible to use eye movement as an index of attention in remote learning scenarios.

It has been shown that eye movement and attention are strictly coupled, both temporally and spatially [13,14]. Thus, observers’ eye movements are often used to investigate the deployment of attention [15,16]. Eye fixations allow people to focus on the target and maintain high acuity of the target on the fovea, and thus can serve as an index of attention [17]. The viewer’s fixational behavior contains abundant spatial information while the transition of fixations includes the temporal information of eye movements. However, most studies investigating the relationships between eye movements and sustained attention emphasized spatial information rather than considering spatial and temporal information jointly. More specifically, most studies analyzed the fixation duration and time points when fixations lie in the pre-defined regions of interest (ROIs) as the indices of attention (e.g., [18,19,20]). Other studies have found an increased number of fixations and longer durations prior to reporting MW in the pre-defined ROIs during a reading task [21,22]. Nevertheless, these studies ignored an important aspect that might provide clues for the relationship between gaze and MW, namely the transition of fixation in the temporal domain.

The transition of fixation in the temporal domain can reflect the planning and strategy processes of the human mind [23] and where the eyes intend to land [24]. Indeed, both are highly correlated with attentional deployment. In addition to attention, individual differences in how likely people will deploy their eye movements to specific regions were identified to be correlated with individuals’ cognitive performances [25]. Yet, this line of approach, by taking both spatial and temporal information of fixations into account, was missing in the research field of sustained attention. In addition, pre-defined ROIs might be subjective and arbitrary in determining the crucial ROIs for detecting sustained attention. Since every researcher has different pre-defined ROIs and different experimental stimuli, when it comes to counting the number of fixations lying in a targeted region (a pre-defined ROI), such as the lecturer in a lecture video, researchers might not be able to generalize the results to other scenarios that do not include a lecturer.

Eye movement analysis with hidden Markov models (EMHMM) can solve such a problem of arbitrarily pre-defined ROIs. This approach can determine ROIs based on transition information between fixations, in addition to fixation locations, and hence provide a data-driven approach for defining the ROIs. In addition, by calculating the probability of transition across ROIs, this approach can take into account both spatial and temporal information as well as individual differences in the viewing paths [26]. For example, Chan et al. [27] discovered that participants who used a similar movement pattern (focusing on the eye region of target faces) to view faces with angry and neutral expressions had higher social anxiety symptoms than those who transitioned their viewing strategies from focusing on the eyes to focusing on the nose. This study showed that relationships between viewing patterns and psychopathology could be revealed by the EMHMM approach. Furthermore, in a face recognition task, Chan et al. [28] found that, in the aging population, those older adults who used more analytic viewing patterns scored higher on their cognitive test performance. Hence, EMHMM can also reveal individual differences in cognitive functions. However, the traditional eye movement data analysis approach cannot achieve these results because the usage of heat maps and fixation counts in pre-defined ROIs can only reveal the frequency that participants focus on the eyes of a face instead of identifying dynamic eye movement patterns. Therefore, by adding the temporal information using EMHMM when determining the relationship between MW and eye movements, we can quantify to what extent participants tend to deploy their eye movements with a specific pattern. This is likely to be related to MW given the close relationship between attention and eye movements. In the near future, this can thus become an index of MW applied to the educational scenario where focused attention is the key to efficient learning [29].

The aim of the current study is to use EMHMM by taking both spatial and temporal information of eye movements to identify specific eye movement patterns that can serve as indices of sustained attention. We hypothesized that MW measured with response to the target (i.e., an objective measure of MW) and subjective report (i.e., a subjective measure of MW) can be revealed by different eye movement patterns.

## 2. Materials and Methods

### 2.1. Participants

The targeted sample size was determined using the effect size (Cohen’s *d* = 1.18) of Chuk et al. [26] where two different viewing patterns in face recognition were found using EMHMM. According to the G-Power 3.1.9.6 software [30], 13 participants for each eye movement viewing group (based on their eye movement patterns; i.e., 26 participants) were required to reach decent statistical power (0.8). To be more conservative, we recruited 20% more participants than needed to verify our results. Therefore, 31 healthy adults were recruited to complete this study (mean age = 22.77 years, SD = 2.87 years, 18 females). All participants were right-handed and free from psychological and neurological disorders. All had normal or corrected-to-normal vision. Participants were naïve to the goal of the experiment. Participants signed the informed consent before the experiment and were rewarded 400 NTD for their participation.

### 2.2. Apparatus and Stimuli

Eye movement data were recorded by Tobii Pro Glasses 2 with Tobii SDK program sampled at 100 Hz. Saccades were defined as eye velocity signals exceeding 100 deg/s, and fixation as events where eye velocity was lower than 100 deg/s and maintained for at least 60 ms.

**Figure 1:**
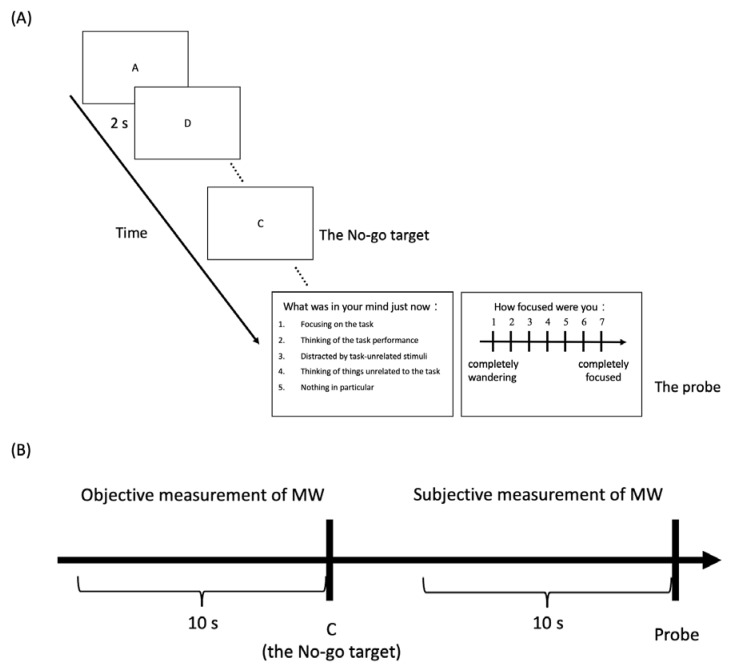
(**A**) Experimental procedure of the SART (the actual background color was grey). Participants were instructed to press the number 8 on the number-pad as quickly as possible whenever they saw an English letter but to withhold their response when they saw the target letter C. After 25 trials (at the end of a block), participants were instructed to answer the two probe questions. (**B**) The analyzed time windows of the SART. The objective measurement of MW was analyzed in the time window 10 s before the English letter C (i.e., the No-go target); the subjective measurement of MW was analyzed in the time window 10 s before the probe.

Stimuli were shown in black against a gray background and presented using the program E-prime (Psychology Software Tools, Pittsburgh, PA, USA). We employed the sustained attention to response task (SART; [31,32]) to measure MW. The SART is a Go/No-go task that has been widely used to induce MW and measure the state of attention. In the SART, 25 English letters (A–Y, except for Z) were presented pseudo-randomly at the center of the screen (extending approximately 0.72° horizontally and vertically), with a target letter (the letter C), presented between the 6th and 15th trial in a block. Each letter was presented for 2 s or until the participant responded. The inter-trial interval (ITI) varied with the reaction time of participants so that each trial (including the ITI) lasted for 2000 ms. For example, if the participant’s reaction time was 300 ms, then the ITI would be 1700 ms to equate the duration of each trial. After 25 trials (with one No-go trial, the target), at the end of each block, participants were asked to answer a probe that asked them to subjectively report their state of attention (Figure 1A). They were instructed to answer the thought probe, “What was in your mind just now?” first with five options consisting of the following: 1. Focusing on the task; 2. Thinking of the task performance; 3. Distracted by task-unrelated stimuli; 4. Thinking of things unrelated to the task; 5. Nothing in particular. Then, participants were asked to rate how focused they were from 1 (completely wandering) to 7 (completely focused) for the moment right before seeing the thought probe. There were 40 blocks in total. Participants were instructed to press 8 on the number-pad to respond to a Go trial and answer the probe question with the corresponding number buttons. After the probe, participants were told to take a short break and press the number 9 to initiate the next block at their own pace.

### 2.3. Procedure

The experiment was conducted in a sound-attenuated room. Participants were seated with their eyes approximately 80 cm away from the monitor and were instructed to do the one-point calibration for the Tobii Pro Glasses 2. Participants were given a detailed description of the thought probe and task content before beginning the experiment. They were told that there was no correct answer regarding the probe so that they could answer truthfully. The main experiment was preceded by three blocks of practice trials (25 trials per block). Participants were required to press a button (i.e., the Go trials) as soon as seeing any English letter other than the letter C, but to withhold a response when the target (i.e., the No-go target, the letter C) was presented. The sensitivity (*d’*) towards the target serves as the main performance index of MW [32,33]. In addition to such an objective measure of MW, we inserted a probe question as the thought sampling method to ask participants what they were thinking about at the moment right before the question appeared. Immediately after the probe question, participants were asked to rate how focused they were from a 7-point Likert scale at the moment right before the probe appeared. Their answer to the probe question and the rating scale about their attentional state (MW or focused, from 1 to 7) were used as the subjective measures of MW. On another note, this study is a portion of a bigger project that includes other physiological measurements, which will not be elaborated here, but see Chen et al. [34].

### 2.4. Data Analysis

We conducted data analysis on the objective and subjective measures of MW separately (Figure 1B). In terms of the objective measure, the 10-s pre-target intervals preceding the No-go target trial were categorized as focused attention (FA) or MW based on the participant’s sensitivity toward the No-go target (*d’*; see below). In terms of the subjective measure, the 10-s pre-probe intervals were categorized as FA or MW based on the participant’s subjective responses to the two probe questions. Subjective FA required fulfilling two criteria, namely responding with options 1, 2, and 5 for the first question (1: Focused on the task, 2: Thinking of the task performance, and 5: Nothing in particular) and having rating scores of 5–7 for the focus rating question on the 7-point scale. Subjective MW also required fulfilling two criteria, namely responding with options 3, 4, and 5 (3: Distracted by task-unrelated stimuli, 4: Thinking of things unrelated to the task, and 5: Nothing in particular) and having rating scores 1–3 for the focus rating question on the 7-point. The 5th option, “Nothing in particular”, was defined as a neutral state and could be considered as either FA or MW, for the following reason. First, given that the SART is a relatively low-demand task, people with high working memory capacity can complete the task by devoting much fewer resources (i.e., nothing in particular in their mind) compared to people with low working memory capacity. Additionally, people tend not to be able to qualify their thought content all the time because it requires the ability to monitor one’s own mental state [35] and might find the other four categories unfit. Thus, we provided the fifth option and categorized the trials selected as “Nothing in particular” as FA or MW based on the response of the subjective rating scale. Since the numeric quantification is more instinctive than content report, trials with focused rating scores greater than 4 were categorized as FA trials, and trials with focused rating scores lower than 4 were categorized as MW trials. Trials with a focused rating score of 4 on the Likert scale were defined as an ambiguous state because people can simultaneously be unfocused but also not mind-wandering (i.e., the gap between MW and FA).

For the objective measure of MW, we quantified participants’ performance of sustained attention based on signal detection theory (SDT) in the time window of 10 s prior to target onset. The 10-s time window was determined according to Christoff et al. [36] who used functional magnetic resonance imaging (fMRI) to reveal the MW-related neural network. A similar time window was also used in other studies (e.g., [8,37]). If participants successfully withheld their response to the target, it was counted as a hit, and if not, the response would be counted as a miss. If participants failed to respond to a non-target letter, it was counted as a false alarm, or it was counted as a correct rejection. We then calculated *d’* based on the hit rate and false alarm rate. With respect to the subjective measure, the proportion of rating FA (out of the 40 probes) was adopted as the dependent variable.

Fixations with durations above three standard deviations of the individual’s mean were excluded (2% for the pre-target session and 2% for the pre-probe session). For the pre-probe intervals, as the rating score 4 is an ambiguous state, either FA or MW, responses with a rating score of 4 were excluded from data analysis (15.81%). Overall, 17.81% of the data were excluded in the pre-probe analysis.

**Figure 2:**
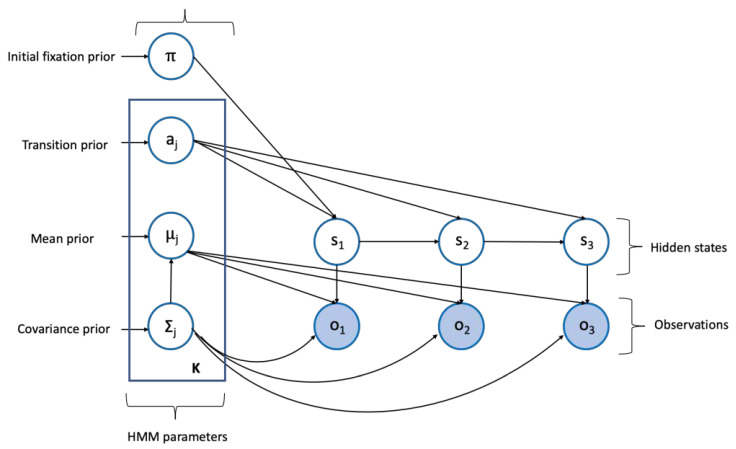
Structure and parameters of an HMM. The O_n_ indicates the observed fixation data; The S_n_ represents the hidden states. The prior distributions of the HMM parameters were presented on the left where K is the number of the hidden states.

Eye movements were analyzed using EMHMM ([26]; toolbox: http://visal.cs.cityu.edu.hk/research/emhmm/ accessed on 1 September 2019). Figure 2 underlines the logic of the EMHMM. The model took the x-y-coordinates for the fixations across time. The time windows of the modeling data were taken from the 10-s pre-target intervals and 10-s pre-probe intervals for objective and subjective measures of MW, respectively. The hidden states of the HMM represented the regions of interest (ROIs) for fixations. Each ROI is a Gaussian, and thus the HMM is a time series of mixtures of Gaussians [38]. We set the possible number of hidden states (ROIs) to be from three (K = 3) to six (K = 6) and chose the one with the highest data log-likelihood using the variational Bayesian method for the pre-target and the pre-probe intervals separately. This allowed us to select the model within this range that had the highest data log-likelihood in a bottom-up (data-driven) way. The parameters of each individual HMM were estimated using the variational Bayesian expectation-maximization (VBEM) algorithm [39], which places a prior distribution on each parameter and then approximates its posterior distribution using a factorized variational distribution.

We then cluster the individuals’ HMMs into groups and form representative HMMs for each group, which summarize each group’s eye movements. The number of clusters was predetermined, which followed previous EMHMM studies where participants’ eye movement patterns could be quantified along the dimension between two contrasting patterns [25,27,28,40,41,42,43,44,45]. To cluster the HMMs into two groups, so as to reveal common patterns among individuals, we used the variational hierarchical expectation-maximization (VHEM) algorithm [46], which clustered HMMs into groups in a bottom-up way based on their similarities and further produced the representative HMMs for each group to describe the ROIs and transitional information in the cluster [26]. More specifically, the algorithm first initialized the Gaussian emissions and transition matrix of each representative HMM using a randomly selected input HMM. Then, it iterated between the E-step and the M-step until convergence. At the E-step, it estimated the expectation of the log-likelihood (similarity) of the representative HMMs with respect to the input HMMs. At the M-step, it grouped the input HMMs according to their similarity to the representative HMMs, and then updated the parameters of the representative HMMs using these cluster assignments [38]. Following previous EMHMM studies, we set the number of ROIs in the representative HMMs to the median number of ROIs in the individual models, performed the VHEM algorithm 100 times, and used the clustering results with the highest expected log-likelihood. We quantified the degree of similarity between individual HMMs and the two representative HMMs using data log-likelihoods. Here, we termed the two representative eye movement patterns distributed and centralized patterns hereafter in the present study based on their characteristics (cf. [26,42,43,45]). The mean-log-likelihood (MLL) of each participant’s eye movement data given the representative HMMs of the distributed and centralized patterns of eye movements was calculated. We defined the D-C scale as the difference in MLL between using the distributed and the centralized patterns [45], which was calculated as the following:

(1) $$\frac{D\;\mathrm{MLL}\;-C\;\mathrm{MLL}}{\left|D\;\mathrm{MLL}\right|+\left|C\;\mathrm{MLL}\right|}$$

where D MLL indicates the MLL given the distributed pattern, and C MLL indicates the MLL given the centralized pattern. A more positive value represents a viewing pattern more similar to the distributed pattern, and a more negative value represents a viewing pattern more similar to the centralized pattern. We then used the D-C scale as a quantitative measure of participants’ eye movement patterns during the task [28].

## 3. Results

### 3.1. Eye Movement Data during the 10-s Pre-Target Period

**Figure 3:**
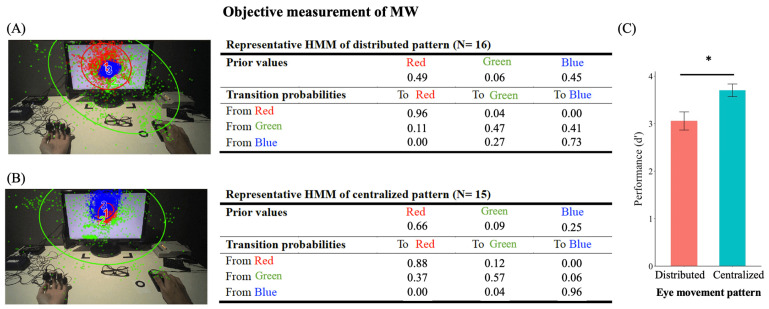
The two representative eye movement patterns during the 10-s pre-target period for (**A**) the distributed eye movement pattern and (**B**) the centralized eye movement pattern. The tables in the middle panel show the transition matrix among the ROIs. Prior values refer to the probability of the first fixation of a trial landed at a specific ROI. (**C**) *d’* of participants with the two eye movement patterns as the objective measurement of MW. Error bars represent one S.E.M. * *p* < 0.05.

The two representative HMMs are shown in Figure 3A,B, which were the distributed pattern and the centralized pattern respectively based on the distributions of their ROIs. To evaluate if the centralized pattern is different from the distributed pattern, we calculated the mean log-likelihoods of the fixation sequences from the distributed pattern using the distributed and the centralized HMMs. The pairwise *t*-test showed that distributed participants’ fixation sequences were more likely to be generated by the distributed HMM than the centralized HMM, *t*(15) = 4.33, *p* < 0.001. The same procedure was used for the centralized group, with similar findings obtained. Namely, centralized participants’ fixation sequences were more likely to be generated by the centralized HMM than the distributed HMM, *t*(14) = 7.63, *p* < 0.001. According to the D-C scale, the distributed pattern (group) consisted of 16 participants and the centralized pattern (group) consisted of 15 participants. Based on the reported ROI locations, orders, and probabilities, people with the distributed pattern had a similar prior probability to start a fixation sequence from the red ROI and the blue ROI, as shown in Figure 3A. They most likely first scanned a wide range across the screen (the red ROI), then looked elsewhere away from the stimuli (the green ROI), and finally scanned back to the central region (the blue ROI). Participants with the centralized pattern most likely focused on the specific central region first (the red ROI), then scanned the left and right sides of the stimuli (the green ROI), and finally returned to the specific central region (the red or blue ROI).

### 3.2. Behavioral Performance during the 10-s Pre-Target Period

**Figure 4:**
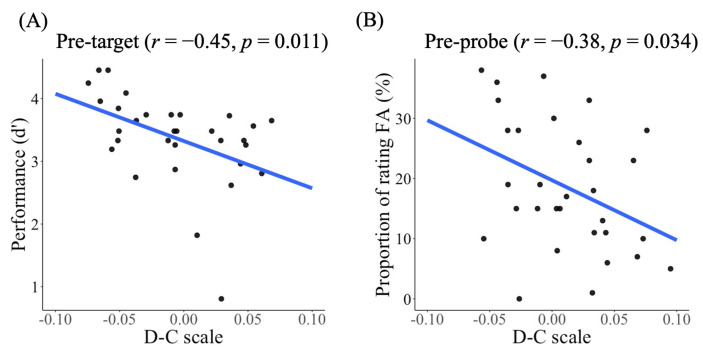
The correlations between (**A**) the performance (*d’*) and the D-C scale during the pre-target phase (**B**) the proportion of rating focused attention (FA) and the D-C scale during the pre-probe phase.

**Figure A1:**
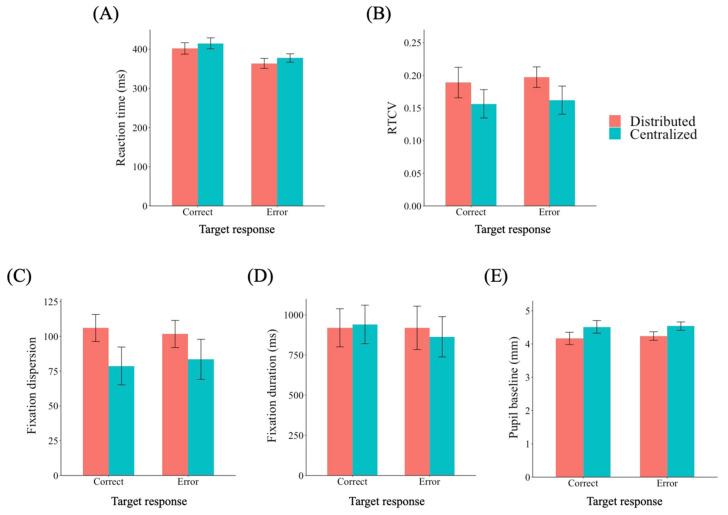
Behavioral performance and eye movement indices in the pre-target intervals. (**A**) Reaction time (RT). (**B**) Coefficient of variance of RT (RTCV). (**C**) Fixation dispersion. (**D**) Fixation duration. (**E**) Pupil baseline. The number in the bar graph denotes the mean value in that condition. Error bars represent one S.E.M.

We compared the task performance using *d’* (see Methods). Figure 3C shows the results of *d’* across groups. Participants with the centralized pattern performed better (i.e., higher *d’*) than those with the distributed pattern, *t*(29) = −2.74, *p* = 0.01, *d* = 0.99. Furthermore, *d’* was negatively correlated with the D-C scale, *r* = −0.45, *p* = 0.011, suggesting that the more distributed the eye movement pattern, the poorer the performance (Figure 4A). Other behavioral performances and eye movement indices are summarized in Appendix A Table A1 and Appendix A Figure A1.

### 3.3. Eye Movement Data during the 10-s Pre-Probe Period

**Figure 5:**
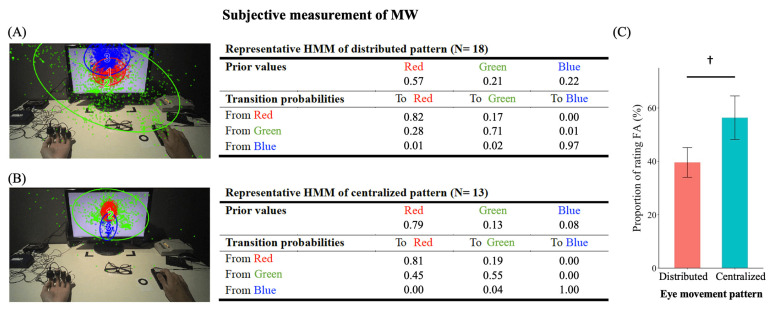
The two representative eye movement patterns during the 10-s pre-probe period for (**A**) the distributed eye movement pattern and (**B**) the centralized eye movement pattern. The tables in the middle panel show the transition matrix. Prior values refer to the probability of the first fixation of a trial landed at a specific ROI. (**C**) The proportion of rating focused attention (FA) for participants with the two eye movement patterns as a subjective measurement of MW. Error bars represent one S.E.M. † *p* < 0.1.

Figure 5A,B show the HMMs of the two representative eye movement patterns. The distributed pattern (group) consisted of 18 participants and the centralized pattern (group) consisted of 13 participants. The pairwise *t*-test showed that distributed participants’ fixation sequences were more likely to be generated by the distributed HMM than the centralized HMM, *t*(17) = 5.79, *p* < 0.001. The same procedure was used on the centralized group, with similar findings obtained. Centralized participants’ fixation sequences were more likely to be generated by the centralized HMM than the distributed HMM, *t*(12) = 5.51, *p* < 0.001. The results here suggest that the distributed and centralized HMMs represent two distinctive eye movement patterns. Based on the reported ROI locations, orders, and probabilities, participants with the distributed pattern demonstrated a wider range of viewing, whereas participants with a centralized pattern showed a high probability to look at the center and continued to view the central region. The ROIs for the centralized pattern were all inside the monitor whereas the ROIs for the distributed pattern were expanded across the entire visual field.

### 3.4. Behavioral Performance during the 10-s Pre-Probe Period

**Figure A2:**
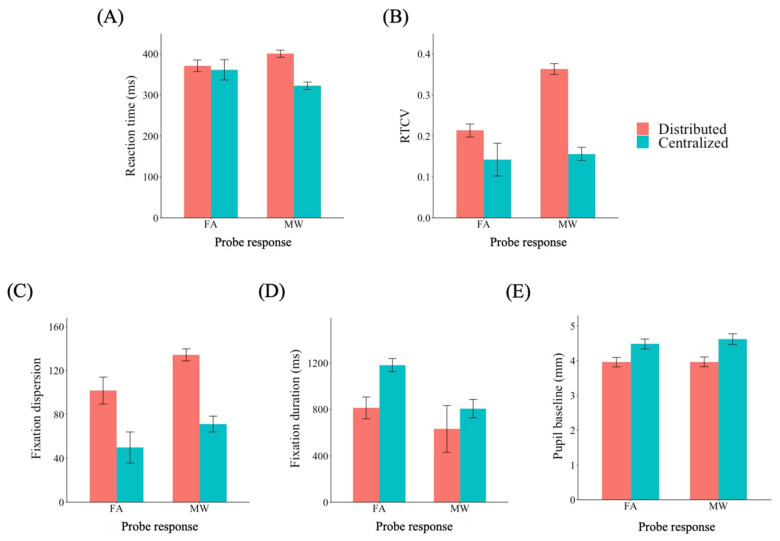
Behavioral performance and eye movement indices in the pre-probe intervals. (**A**) Reaction time (RT). (**B**) Coefficient of variance of RT (RTCV). (**C**) Fixation dispersion. (**D**) Fixation duration. (**E**) Pupil baseline. The number in the bar graph denotes the mean value in that condition. Error bars represent one S.E.M. FA: Focused attention. MW: Mind-wandering.

We compared the task performance of participants using the two eye movement patterns in the proportion of rating FA. Participants with the centralized pattern tended to rate themselves as more focused than those with the distributed pattern, t(29) = −1.76, *p* = 0.089, *d* = 0.629 (Figure 5C). The proportion of rating FA was negatively correlated with the D-C scale, *r* = −0.38, *p* = 0.034, suggesting that the more distributed the pattern, the lower the proportion of self-rated FA (Figure 4B). Other behavioral performance data and eye movement indices were summarized in Appendix A Table A2 and Appendix A Figure A2.

### 3.5. Trial by Trial Analysis

To examine if our model can work on a trial-by-trial level instead of being limited to classifying people who are more prone to MW from those who are not, following Zhang et al. [44], the eye movement pattern for each trial across all participants was classified into the centralized or distributed pattern according to the log-likelihood generated by the representative model (Appendix A Table A3 and Table A4). Here, we performed the likelihood ratio chi-squared statistical analysis (the *G*^2^ test) to see if trials belonging to the centralized pattern would be more likely to have correct no-responses to the target and also have higher proportions of rating FA. The *G*^2^ test is a maximum likelihood statistical test that provides an approximation of the theoretical chi-squared distribution better than the Pearson’s chi-squared test [47]. For pre-target intervals, the odds ratio for trials in the centralized pattern to withhold successfully and trials in the distributed pattern to fail to withhold were 1.88 times more likely than vice versa (*G*^2^ = 24.01, *p* < 0.001). For the pre-probe intervals, the odds ratio given that trials in the centralized pattern were scored as FA and trials in the distributed pattern were scored as MW was 2.39 times more likely than vice versa (*G*^2^ = 33.56, *p* < 0.001). The results support that the eye movement pattern measures quantified using EMHMM are not just limited to the participants’ trait level, but can also be applied trial-by-trial. Namely, by analyzing distributed trials and centralized trials, we can apply the model to a trial-by-trial basis. Therefore, we are able to instantly detect people’s attentional state based on the eye movement pattern in real-time rather than only classifying attentional states based on subsequent data analyses.

## 4. Discussion

The current study found that MW can be revealed by eye movement patterns, as we categorized eye movement patterns into the distributed pattern and the centralized pattern via EMHMM. More importantly, we discovered that participants with the distributed pattern were more prone to MW than people using the centralized pattern. We drew this conclusion after analyzing an objective measure regarding performance towards the target and subjective ratings that included lower sensitivity (*d’*) towards the target (withholding keypress) and a lower proportion of rating FA.

### 4.1. The Relationships between Mind-Wandering and Eye Movement Patterns

In line with the discoveries in the current study, people who used a more centralized (less distributed) eye movement pattern as their strategy had better cognitive performances. For example, Chan et al. [28] found that older adults whose eye movement patterns showed better concentration on facial features in a face recognition task (i.e., the analytic pattern) had higher scores in the Montreal Cognitive Assessment (MoCA), which is a well-established neuropsychological test examining people’s language, executive, visuospatial processing, and memory functions [48]. Chan et al. [49] also showed that people using the analytic pattern demonstrated more activation in brain regions related to top-down control compared to people using the holistic viewing pattern in a face recognition task, such as the frontal eye field (FEF), dorsolateral prefrontal cortex (DLPFC), and intraparietal sulcus (IPS). Therefore, it is possible that participants who adopted the centralized pattern engaged more top-down control of attention that helped filter out irrelevant information and improved the efficiency of information processing [49]. Additionally, in the education scenario, Zheng et al. [45] showed that participants who looked more at the center of the screen (i.e., the centralized pattern) had better comprehension of the lesson materials than those who looked around more (i.e., the distributed pattern). These studies along with our results verified that people using the centralized pattern have better cognitive performance in general compared to people using the distributed pattern.

In addition, both objective and subjective indices (*d’* and proportion of rating FA) were negatively correlated with the D-C scale, suggesting that the more distributed the eye movement pattern, the worse the performance in both measurements. The likelihood ratio test further verified that trials with the centralized pattern were more likely to be FA and trials with the distributed pattern were more likely to be MW. Therefore, in the future, we could possibly replace task performance index and subjective reports of attentional states with eye movement behaviors as a real-time indicator of MW. To be more specific, if the distributed pattern is detected from one’s eye movements, there is a high possibility of disengagement from one’s current task. As sustained attention plays a critical role in learning and memory [50], understanding when people tend to mind-wander may help them direct their focused attention back to the learning materials.

Some may argue that the SART commission error and probe-based response cannot represent the MW state. For instance, looking at the commission error as the objective measurement of MW, Head and Helton [51] suggested that the commission error indicates the failure of executive control but not MW. However, according to Robertson et al. [32], the failure of executive control is the “consequence” of MW but not the main cause of the commission error. Indeed, Seli [52] has shown that SART errors do reflect MW even when controlling the RTs for go trials. As for the probe-based responses, despite some studies questioning its validity (e.g., [53]) and its characteristic to interrupt the task [54], others have proposed that thought probe is relatively robust to the variability of task parameters, and hence suitable to examine the thought content during a MW-related task [55]. Notwithstanding the limitations of the thought probe response, we investigated the MW based on the objective and subjective measure as Faber et al. [56] suggested, and found similar eye movement pattern for objective and subjective MW (i.e., the distributed pattern) as well as objective and subjective FA (i.e., the centralized pattern).

### 4.2. Applications and Future Works

The eye movement detection system for MW can be applied to educational scenarios. Indeed, the commission error in the SART is a sensitive measure of the attention that is associated with the focused state of children during class [57]. Previous studies using behavioral tasks to measure MW, such as a detection system that pops out a window to check students’ attentional states after 10 min of idle time (when no mouse movement or keyboard activity has been detected), might interfere with the learning process. Such a method of monitoring student performance is disturbing and may cause dual-task interference (i.e., taking notes and moving the mouse), which leads to a more significant cognitive overload and might not be the best way to capture students’ attentional states [58]. Using an eye-tracking system to detect MW can thus avoid causing the extra dual-task demand. More importantly, the SART here is essentially analogous to the scenario when we are listening to lectures. Imagine that we are in a lecture, we are more likely to miss the content that the lecturer refers to when our mind wanders, which is parallel with the commission error found in the SART (i.e., skipping the No-go target). Meanwhile, we tend to start to retrospectively evaluate our own states and our understanding of the lecture, specifically when the lecturer calls on us during class. This phenomenon is similar to the probe question used in our task. To sum up, we consider the task we used here suitable to examine the states of attention and thus it can be applied to the educational scenario. We expect that eye movement patterns can assist teachers in observing inattentive behaviors directly in the classroom without interfering with students’ learning.

We have shown that people with the distributed pattern are more prone to MW compared to people with the centralized pattern, either with objective or subjective measures. As the centralized pattern is associated with being focused and having more top-down control of attention, future studies can develop a training program to examine if altering people’s viewing pattern from the distributed pattern to the centralized one can enhance cognitive ability and thus task performance. Not only can this help people who are easily distracted, but it can also aid older adults in performing cognitive-demanding tasks, as older adults tend to use the distributed pattern for cognitive processes [28]. This was not revealed by previous studies using traditional eye movement indices to analyze or classify MW state [59]. Specifically, Faber et al. [59] proposed that the eye movement pattern indices associated with MW might vary across tasks and that fixation is not a robust index for MW in centralized tasks, such as the SART used here and other audiobook tasks. In contrast, we demonstrated that when combining the transition matrix of eye movement in the temporal domain, fixation can still effectively predict the mental state, either being MW or FA. Furthermore, as MW during long-term driving occurs very often and can cause negative influences on safety [8,60], future developers can consider installing an eye movement pattern detection system in a car. With the aid of the detection system, drivers can reacquire the FA state (centralized pattern) whenever the distributed pattern is detected. Future studies can further examine if this pattern also applies to tasks with different spatial, visual, or discourse demands and other modalities (e.g., auditory stimuli) [61].

What is the benefit of using HMM rather than the deep neural net (DNN) or other alternative approaches to detect MW? Since HMM is a probabilistic time-series model, it works well with a limited amount of data, which is in contrast to deep learning methods that require large amounts of data to train the model effectively. In addition, when there is a large pool of participants, learning individual models can be done in parallel, which makes it scalable. Furthermore, the VHEM algorithm for clustering HMMs is based on the parameters of the individual HMMs instead of the actual data, and the clustering can be done efficiently, too. For alternative learning methods, as compared with the recurrent DNN for predicting sequential information, one advantage of using HMMs is that it can make the learning model(s) more interpretable, which is an important trend in the current artificial intelligence research (e.g., [62,63]). For example, we have recently developed a computational model that combines a DNN with an HMM to learn eye movement strategies for object recognition [64]. The DNN learns optimal perceptual representations under the guidance of an attention mechanism summarized in an HMM, and the HMM learns optimal eye movement strategies through feedback from the DNN. The resulting HMM of the model is immediately interpretable and can be directly used for data analysis.

## 5. Conclusions

Our results suggest that eye movement patterns are associated with MW, where both objective and subjective measures of MW can be distinguished from focused attention state by the viewer’s more distributed eye movement pattern. The current study is important both technically and practically. First, we provide a novel approach to utilizing EMHMM to study sustained attention. Second, we show that eye movements can be a potential way to detect people’s state of attention, which can be used in either in-person or remote classes so that instructors can have better ideas about students’ attention states and find ways to regain their attention once MW is detected.

## Data Availability

The data in this study are available from the link: https://osf.io/zy3v7/?view_only=3075989e8eb140a8ab7d1ac7cb409887.

## Appendix A

To examine the differences between the two distinct eye movement patterns in behavioral performance and eye movement indices, we used the lme4 package in R [65] to conduct linear mixed-effect models (LMM) for explorative data analysis. To the non-targets (Go trials), we analyzed the mean reaction time (RT). To the targets (the No-go targets) and the probes, we analyzed the coefficient of variance of RT (RTCV) from the 10-s time windows prior to the presentation of the target and the probe, respectively. For calculating pupil baseline, the data was first down-sampled from 100 Hz to 50 Hz, then the data from the left and right eyes were averaged. Lastly, the pupil baseline was defined as the average pupil diameter 500-ms before the onset of each stimulus.

In the LMM, the target response or the probe response and the eye movement patterns served as fixed effects, and participants served as a random effect. The approximation of the *p*-values came from the lmerTest package.

For the pre-target period (Table A1 and Figure A1), slower RTs to Go-stimuli (*t* = 6.7, *p* < 0.001) and a smaller pupil baseline (*t* = −2.29, *p* = 0.029) were found in the correct versus error target response (i.e., response to the No-go targets), which was in line with previous studies [34,66,67]. No other effects were found, *ps* > 0.05.

For the pre-probe period (Table A2 and Figure A2), a significant interaction between probe response and eye movement pattern was found in RTCV (*t* = −2.58, *p* = 0.015). Post-hoc analysis revealed that in the self-rated FA condition, participants with the centralized pattern showed smaller RTCVs than those with the distributed pattern, *t* = 3.15, *p* = 0.004. In addition, participants with the centralized pattern also showed smaller fixation dispersion (*t* = −3.11, *p* = 0.003), longer fixation duration (*t* = 2.16, *p* = 0.037), and a larger pupil baseline (*t* = 2.38, *p* = 0.024) compared to participants with the distributed pattern. These results suggested that people with the centralized pattern tended to pay more attention overall. Additionally, the fixation dispersion was smaller when participants rated themselves as FA compared to MW (*t* = 2.73, *p* = 0.012).

**Table A1:** Behavioral response data and eye movement data for the 10-s pre-target period (the objective measurement of MW) analyzed with LMM.

	Estimate	SE	*t*	*p*
**RT**				
(Intercept)	363.83	12.94	28.12	<0.001 ***
Target response	38.12	6.63	5.70	<0.001 ***
Eye movement pattern	13.78	18.60	0.74	0.464
Target response × Eye movement pattern	−0.74	9.62	−0.08	0.939
**RTCV**				
(Intercept)	0.20	0.02	9.57	<0.001 ***
Target response	−0.01	0.02	−0.55	0.590
Eye movement pattern	−0.04	0.03	−1.19	0.243
Target response × Eye movement pattern	0.003	0.02	0.12	0.908
**Fixation dispersion**				
(Intercept)	101.72	11.87	8.57	<0.001 ***
Target response	4.29	8.43	0.51	0.615
Eye movement pattern	−18.26	17.07	−1.07	0.291
Target response × Eye movement pattern	−9.06	12.12	−0.75	0.461
**Fixation duration**				
(Intercept)	892.75	123.08	7.25	<0.001 ***
Target response	26.60	33.22	0.80	0.430
Eye movement pattern	−29.22	176.94	−0.17	0.870
Target response × Eye movement pattern	50.28	47.75	1.05	0.301
**Pupil baseline**				
(Intercept)	4.24	0.16	26.45	<0.001 ***
Target response	−0.07	0.03	−2.29	0.029 *
Eye movement pattern	0.30	0.23	1.31	0.201
Target response × Eye movement pattern	0.05	0.04	1.05	0.302

* *p* < 0.05; *** *p* < 0.001.

**Table A2:** Behavioral response data and eye movement data for the 10-s pre-probe period (the subjective measurement of MW) analyzed with LMM.

	Estimate	SE	*t*	*p*
**RT**				
(Intercept)	363.27	17.81	20.40	<0.001 ***
Probe response	21.07	17.03	1.24	0.229
Eye movement pattern	−3.69	27.92	−0.13	0.896
Probe response × Eye movement pattern	−58.81	30.49	−1.93	0.065
**RTCV**				
(Intercept)	0.24	0.03	8.17	<0.001 ***
Probe response	0.17	0.03	5.18	<0.001 ***
Eye movement pattern	−0.09	0.05	−1.97	0.055
Probe response × Eye movement pattern	−0.15	0.06	−2.58	0.015 *
**Fixation dispersion**				
(Intercept)	101.73	10.70	9.51	<0.001 ***
Probe response	30.52	11.16	2.73	0.012 *
Eye movement pattern	−52.25	16.80	−3.11	0.003 **
Probe response × Eye movement pattern	−5.94	19.50	−0.31	0.763
**Fixation duration**				
(Intercept)	813.67	108.01	7.53	<0.001 ***
Probe response	−192.01	106.36	−1.81	0.087
Eye movement pattern	365.86	169.40	2.16	0.037 *
Probe response × Eye movement pattern	−211.32	186.65	−1.13	0.270
**Pupil baseline**				
(Intercept)	3.96	0.13	30.16	<0.001 ***
Probe response	0.09	0.06	1.36	0.189
Eye movement pattern	0.48	0.20	2.38	0.024 *
Probe response × Eye movement pattern	0.11	0.12	0.97	0.344

* *p* < 0.05; ** *p* < 0.01; *** *p* < 0.001.

**Table A3:** Trial numbers across conditions in the objective measure of MW (response to the No-go target).

	Centralized Pattern	Distributed Pattern
Correct	514 (58.68%)	362 (41.32%)
Error	144 (42.99%)	191 (57.01%)

*Note.* Numbers in the table indicate trials that belonged to Correct (successful stop) or Error (fail-to-stop) responses to No-go targets, which were classified as centralized or distributed patterns based on the model. The numbers in the parathesis indicate the proportion of trials that belonged to either the centralized or the distributed pattern in the correct and error responses respectively.

**Table A4:** Trial numbers across conditions in the subjective measure of MW (response to the probe).

	Centralized Pattern	Distributed Pattern
Self-rated FA	329 (55.02%)	269 (44.98%)
Self-rated MW	90 (33.83%)	176 (66.17%)

*Note.* Numbers in the table indicate trials that belong to self-rated FA or MW, which were classified as centralized or distributed patterns based on the model. The numbers in the parathesis indicate the proportion of trials that belonged to the centralized or the distributed pattern in the self-rated FA and self-rated MW respectively.
